# Ultrasound-Guided Multilevel Bilateral Rectus Sheath Blocks with Liposomal Bupivacaine, Bupivacaine, and Dexamethasone PF for Postoperative Pain Management After a Pediatric Abdominal Aortic Aneurysm Repair

**DOI:** 10.3390/children12111450

**Published:** 2025-10-25

**Authors:** Tyler H. Augi, Mihaela Visoiu

**Affiliations:** 1 School of Medicine, University of Pittsburgh, Pittsburgh, PA 15261, USA; tha25@pitt.edu; 2 Department of Anesthesiology and Perioperative Medicine, UPMC Children’s Hospital of Pittsburgh, School of Medicine, University of Pittsburgh, Pittsburgh, PA 15213, USA

**Keywords:** abdominal aortic aneurysm, rectus sheath block, liposomal bupivacaine, regional anesthesia, pediatric, acute pain

## Abstract

**Highlights:**

**What are the main findings?**

• Ultrasound-guided multilevel bilateral rectus sheath blocks utilizing a combination of liposomal bupivacaine, bupivacaine, and dexamethasone PF provided prolonged, opioid-sparing analgesia following open pediatric AAA repair.
• There was no evidence of local anesthetic systemic toxicity observed in association with this analgesic approach.

**What is the implication of the main finding?**

• This multilevel rectus sheath block technique (using liposomal bupivacaine, bupivacaine, and dexamethasone PF) may represent a safe and effective alternative to neuraxial techniques for complex pediatric abdominal surgery.
• It is particularly relevant for patients in whom anticoagulation therapy or the need for neurologic monitoring makes epidural or paravertebral blocks a high-risk option.

**Abstract:**

**Background/Objectives**: Abdominal aortic aneurysms (AAAs) are exceedingly rare in pediatric patients but carry a significant risk of rupture, necessitating urgent surgical repair. Postoperative pain management following open AAA repair is particularly challenging and ultrasound-guided rectus sheath blocks (RSBs) offer a targeted and lower-risk alternative for midline abdominal incisions. **Methods**: We present an 8-year-old male who underwent open infrarenal AAA repair. Multilevel bilateral ultrasound-guided RSBs were performed at T7, the umbilicus and T12 using a mixture of liposomal bupivacaine, bupivacaine, and dexamethasone preservative free (PF). **Results**: Postoperative pain scores remained consistently low through postoperative day (POD) 6, with minimal opioid requirements. Functional recovery was rapid, with sitting achieved by POD 1 and ambulation by POD 2. Plasma bupivacaine concentrations remained within safe limits throughout hospitalization. **Conclusions**: Multilevel bilateral RSBs with liposomal bupivacaine, bupivacaine, and dexamethasone PF provided prolonged opioid-sparing analgesia, facilitated early mobilization, and supported enhanced recovery in this complex pediatric surgical case.

## 1. Introduction

Abdominal aortic aneurysms (AAAs) are sporadic among children, with few cases documented in medical literature. In pediatric cases, the open surgical approach is often preferred to ensure durable long-term results and accommodate growth [1,2,3]. Open AAA repair can be complicated by significant post-operative pain, which may result in prolonged immobility and difficulty clearing the airways. These issues can contribute to poorer outcomes, including extended hospital stays and an increased risk of pulmonary complications [4,5]. Given these complications, effective post-operative pain management is essential.

The approach to pain control following open AAA repair has evolved from relying solely on epidural analgesia to incorporating alternative methods, such as rectus sheath blocks (RSBs) [6]. Epidural analgesia carries risks, including bleeding complications, particularly in patients who require perioperative anticoagulation. In contrast, RSBs have a much lower risk profile [6]. Studies have shown that RSBs can significantly reduce opioid consumption and enhance pain control in patients undergoing major abdominal surgeries, including open AAA repair. For instance, Cleary et al. [7] found that patients who received rectus sheath blocks (RSBs) had a shorter time to extubation, reduced postoperative opioid requirements, and shorter hospital stays compared to those who received only general anesthesia.

Liposomal bupivacaine, a novel sustained-release formulation of bupivacaine hydrochloride, has been increasingly utilized for postoperative pain management due to its extended duration of action. This formulation encapsulates bupivacaine within multivesicular liposomes, allowing for a prolonged analgesic effect lasting up to 72 h post-administration [8,9]. The use of liposomal bupivacaine has been shown to reduce the need for opioid analgesics, thereby minimizing opioid-related adverse events and enhancing patient recovery [9,10]. In the pediatric population, although liposomal bupivacaine is not yet FDA-approved, its off-label use has been explored with promising results in terms of safety and efficacy [11,12,13].

This case report describes the use of multilevel, bilateral ultrasound-guided rectus sheath blocks (bURSBs) with liposomal bupivacaine, bupivacaine, and dexamethasone preservative free (PF) in an 8-year-old patient undergoing open AAA repair. It highlights this approach as a potentially effective and safe alternative to neuraxial anesthesia, as part of a multimodal approach, in high-risk pediatric surgical patients.

## 2. Case Description

Special approval was obtained from the patient and his family, the pharmacy, and the vascular surgeon to use liposomal bupivacaine off-label for a peripheral nerve block in this pediatric patient. The patients’ parents gave written informed consent for the presentation of this report.

The patient was an 8-year-old male (131.5 cm, 26.7 kg), with no medical history, except migraines, who was initially seen in the emergency department after his pediatrician discovered a left brachial pseudoaneurysm. Further evaluation revealed several aneurysms, including a large infrarenal aortic aneurysm measuring 4.5 cm. Given the size and risk of rupture, vascular surgery was recommended to repair the AAA. Later genetic testing was normal, and an MRI revealed a left petrous segment internal carotid artery aneurysm.

Ultrasound-guided, multiple-level bilateral rectus sheath blocks with liposomal bupivacaine, bupivacaine, and dexamethasone PF were chosen for postoperative pain control. Approval was explicitly granted for this patient, as it was anticipated that administering liposomal bupivacaine in the rectus sheath blocks would provide effective pain control for several days. The decision was supported by previous studies demonstrating the safety of liposomal bupivacaine when administered via infiltration in pediatric patients aged six years and older [8]. Moreover, the pain specialist had prior experience using this medication for pediatric nerve blocks.

**Figure 1:**
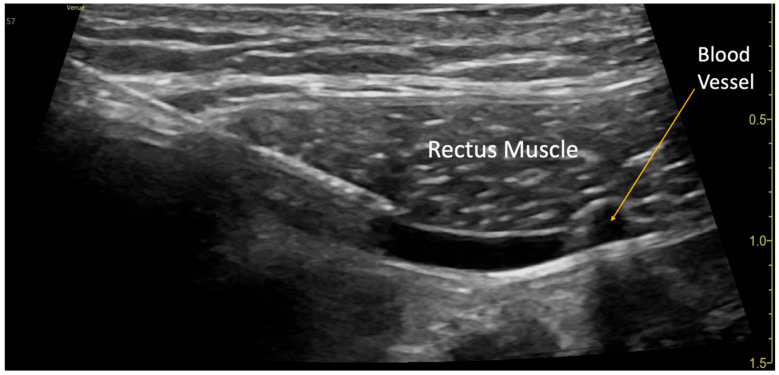
Rectus Sheath Block Procedure. A blood vessel was visualized in the plane underneath the rectus muscle.

**Figure 2:**
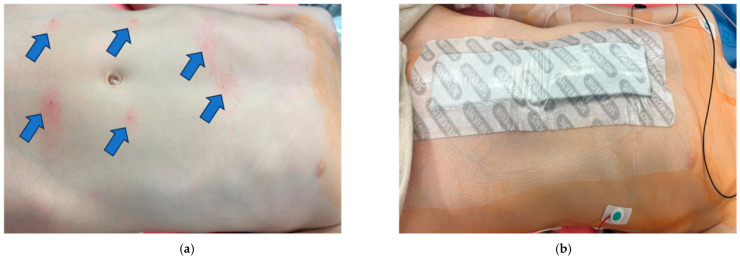
(**a**) Location of the rectus sheath blocks with arrows pointed to the specific injection sites, bilateral T7, T10, and T12 dermatomes; (**b**) Bandage covering the surgical incision site, extending from the xyphoid process to the pubic symphysis.

**Figure 3:**
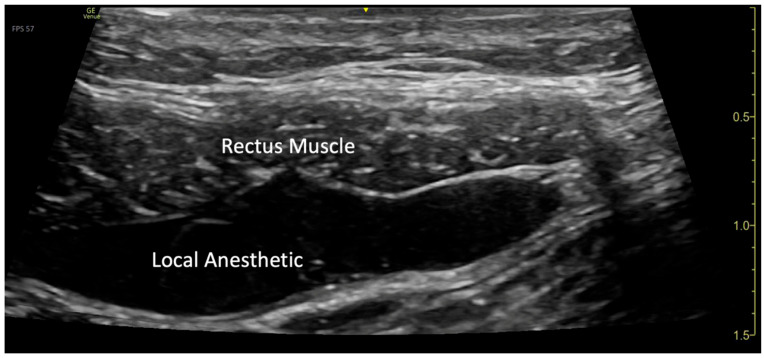
Visualization of local anesthetic spread under the rectus muscle.

The blocks were performed while the patient was under general anesthesia, before surgery started, and after sterile preparation. A high-frequency linear transducer was positioned at the level of the umbilicus and moved laterally a few centimeters until the lateral border of the rectus muscle was visualized. An echogenic needle, 22-gauge, 50 mm Sono-TAP (Pajunck Medical Inc., Geisingen, Germany) was advanced medially from the lateral edge of the probe; final needle placement was at the lateral border of the rectus muscle, deep to the muscle, yet superficial to the posterior aspect of the rectus sheath, and not pre-peritoneal nor intraabdominal. A few milliliters (mL) of saline solution were injected until the spread of medication was visualized in the correct location (Figure 1), followed by three milliliters (mL) of local anesthetic mixture (liposomal bupivacaine 113.05 mg, bupivacaine 0.25%, 32.5 mg, and dexamethasone PF, 5 mg-total volume 22 mL). This procedure was repeated at the contralateral site. Two more bilateral local anesthetic injections under the rectus muscle were performed at the level of T7 and T12 dermatomes (Figure 2a). A total of six rectus sheath blocks were performed with 22 mL of local anesthetic mixture. After the blocks were performed, medication spread behind the rectus muscle was visualized as a bulge into the space under the rectus sheath muscle from the xiphoid to the pubic symphysis (Figure 3), as described by Visoiu et al. [14], matching the incision performed (Figure 2b) Video S1. The surgery then conducted was an open infrarenal abdominal aortic aneurysm repair with a Hemashift Gold 12 mm graft and a 6 mm limb used for aortic graft to common iliac aneurysm repair. The duration of anesthesia was 374 min, the aortic cross-clamp time was 95 min, and total blood loss for the operation was 500 mL with 180 mL of blood salvage. He also received 4500 units of Heparin during the surgery, which was reversed with 10 mg of protamine. The surgery was uneventful, and intraoperative hemodynamic stability was adequately maintained. The patient was extubated in the operating room.

Intraoperative medications included intravenous (IV) fentanyl 2.25 mcg/kg, IV methadone at 0.22 mg/kg, IV ketamine at 1.87 mg/kg and IV acetaminophen at 14.98 mg/kg. Postoperative medications can be found in Table 1. The patient was discharged in stable condition on POD 6 with acetaminophen 400 mg to take as needed for pain control and aspirin 81 mg daily.

The ketamine administration resulted in the patient experiencing double vision and hallucinations on POD 0 and POD 1. Ketamine was discontinued on POD 1, which resulted in the resolution of side effects. He did develop headaches following the discontinuation of ketamine, which were alleviated through cold packs.

Postoperative pain scores were collected using the patient reported Visual Analog Scale and remained consistently low throughout the hospital stay. The patient reported no pain on postoperative day (POD) 0. The average pain score, which was determined as the average of all reported scores over each POD, was 0.5 out of 10 on POD 1, with a highest reported score of 6. By POD 2, pain decreased further to an average of 0.29 with a maximum of 2, and remained low on PODs 3 (average 0.4, max 2), 4 (average 1, max 7), and 5 (average 0.29, max 2). The patient reported no pain on POD 6. The maximum pain scores on POD 2 and 3 were attributed to headaches, while those on POD 0, 1, 4, and 5 were attributed to post-operative incisional pain at the upper area part of the incision, an area that did not have any numbness at that time.

Functional recovery milestones were achieved promptly. The patient was initially bedbound on POD 0 but was able to get out of bed to a chair by POD 1. Ambulation began on POD 2 and continued through POD 6. There was a concern for post-operative ileus, and the patient was nil per os (NPO) for 48 h post-op, but this concern resolved with the patient tolerating liquids on POD 3, solids on POD 4, and having a bowel movement on POD 4. Complete pain scores and functional recovery milestones can be found in Table 2.

Plasma bupivacaine levels were measured on PODs 1 through 3 and remained within safe limits: 0.11 mcg/mL on POD 1, 0.093 mcg/mL on POD 2, and 0.11 mcg/mL on POD 3.

Numbness on each side of the body could not initially be investigated due to the large dressing covering the incision area. By POD 5, the patient reported bilateral numbness along portions of the incision, which resolved one day later.

## 3. Discussion

This case demonstrates the successful use of ultrasound-guided multilevel bilateral rectus sheath blocks (RSBs) with bupivacaine, liposomal bupivacaine, and dexamethasone PF for postoperative pain management in an 8-year-old patient undergoing open abdominal aortic aneurysm (AAA) repair—a rare and complex surgical procedure in the pediatric population.

In this challenging case, multilevel bilateral ultrasound guided RSBs were chosen as the most appropriate regional anesthesia for postoperative pain management. Before surgery, we evaluated several other regional pain control options, but each had significant drawbacks. An epidural catheter was considered; however, due to the patient’s multiple aneurysms, we were concerned about the possibility of an aneurysm in the epidural space. Additionally, we anticipated the need for anticoagulation both during and after surgery, as well as potential postoperative intubation, which could complicate the neurological examination of the extremities. Paravertebral catheters were another option but posed similar risks to the epidural approach. Bilateral rectus sheath catheters were considered; however, we were concerned that they would not provide adequate analgesia for such a large incision, as the maximum volume of local anesthetic deliverable through each catheter would be limited to 3.4 mL/h. Moreover, these catheters would need to be placed at the end of surgery, as they would be near the surgical incisions.

Transversus abdominus plane (TAP) blocks were not considered for this operation as this approach provides only lower abdominal sensory blockade in children, usually of three to four dermatomes. Such blocks should be reserved for lower abdominal surgery, under the umbilicus, as only 25% of patients in a study performed by Palmer et al. [15] had upper abdominal block extension. Furthermore, combining TAP blocks with subcostal TAP blocks could be considered; however, this approach would require a higher total dose of local anesthetic and would limit the ability to assess its spread at the target dermatome levels. Furthermore, there are no case reports of sing TAP blocks with liposomal bupivacaine in pediatric patients to provide guidance on technique.

Rectus sheath blocks (RSBs) have proven effective in reducing postoperative opioid consumption, facilitating early extubation, and promoting early mobilization and discharge [6,7]. For example, Cleary et al. [7] reported that RSBs, with or without catheter insertion, performed for pain control after adult aortic aneurysm surgeries, resulted in shorter extubation times, reduced opioid use, and decreased hospital stays compared to general anesthesia alone. The blocks were performed at a single level, with a high volume of diluted, short-lasting local anesthetic.

In our case, we expected that blocks performed at the level of the umbilicus only would not provide adequate analgesia across the entire incision, as noted by Visoiu et al. [14]. Therefore, we performed additional bilateral rectus sheath blocks both above and below the umbilicus. Using ultrasound guidance, we were able to visualize the needle placement and medication spread from the xiphoid to the pubis, ensuring effective pain relief across the entire surgical incision.

The patient’s postoperative recovery supports the efficacy of the analgesic approach. To expedite the onset of the nerve block, 1.28 mg/kg of plain bupivacaine was co-administered with liposomal bupivacaine. Given the lack of clear dosage recommendations for liposomal bupivacaine, particularly in the context of ultrasound-guided fascial plane block, we elected to administer 4.23 mg/kg (8.5 mL), to prolong the duration of analgesia. This dose approximates the pediatric recommendation for liposomal bupivacaine when used for wound infiltration. This medication, although not FDA-approved for pediatric use, has demonstrated extended analgesia due to its sustained-release properties, lasting up to 72 h post-injection; warranting further research to establish evidence-based pediatric dosing guidelines [8,9,10,13].

To further prolong pain relief, we added dexamethasone PF to the blocks. For our patient, pain scores at the abdominal incision remained consistently low throughout the hospitalization, with no incisional pain reported by POD 6. It is important to note that the pain scores were in line with the sensory exam findings, which indicated numbness on POD 5, and the presence of bupivacaine in the blood on POD 3. Plasma bupivacaine levels remained well within the safe range, and no signs of local anesthetic systemic toxicity were observed. Monitoring bupivacaine levels allowed us to correlate the duration of analgesia with both the presence of numbness and the anesthetic’s blood concentration. This correlation further validates the effectiveness of the pain management strategy and the prolonged analgesia provided by the block. It is possible that dexamethasone PF contributed to the prolonged numbness (until POD 5) and extended analgesic effect through its anti-inflammatory properties and local anesthetic-sparing effects [11,12]. A recently published trial also found that dexamethasone PF prolonged liposomal bupivacaine’s duration of analgesia and reduced opioid use compared to liposomal bupivacaine alone when used for local infiltration in total shoulder arthroplasty patients, providing further support for our clinical findings [16]. The patient reported mild numbness at the incision site by POD 5, which lasted longer than previously reported in the case report, where dexamethasone PF was not included in the nerve blocks [13].

The multimodal analgesic regimen for this patient included scheduled acetaminophen, intermittent methadone doses, a short course of ketamine and ketorolac, and minimal use of short-acting opioids—highlighting the opioid-sparing effect of the block. This approach successfully minimized opioid exposure, which facilitated the resumption of bowel function and helped prevent ileus, a potential complication after surgery [17]. Early mobilization was achieved, with the patient sitting up by POD 1 and ambulating by POD 2.

The patient, family, and surgical team all expressed increased satisfaction with the pain management approach. A few months later, the patient underwent a nephrectomy due to an unresectable aneurysm of the renal artery. For this procedure, the family requested nerve blocks with liposomal bupivacaine for postoperative pain management.

Liposomal bupivacaine’s sustained-release properties, potentiated by dexamethasone PF’s anti-inflammatory and analgesic synergy, may offer prolonged, safe, and effective regional analgesia, without the need for the placement of catheters. Investigating optimal dosing, safety, and duration of effect of this medication combination in rectus sheath blocks could facilitate the development of protocols that minimize systemic opioid exposure, reduce the risks associated with central neuraxial techniques, and enhance postoperative recovery and patient comfort in high-risk pediatric surgical populations.

While this patient had a positive outcome, there are limitations that come with this case report. First, since this is only one patient’s case there is a lack of comparison or control group. We also used liposomal bupivacaine off-label with no randomized controlled study proving its efficacy for this exact indication. Finally, given that this is a case report we acknowledge that these findings may not be generalizable to the general population at large. While these limitations are apparent, this unique case provides a valuable addition to the literature.

## 4. Conclusions

This case illustrates the successful use of ultrasound-guided multilevel bilateral rectus sheath blocks (RSBs) with liposomal bupivacaine, bupivacaine, and dexamethasone PF for postoperative pain management in a pediatric patient undergoing open abdominal aortic aneurysm repair. The approach effectively minimized opioid use, reduced the risk of complications such as ileus, and facilitated early extubation and mobilization. The addition of liposomal bupivacaine, bupivacaine, and dexamethasone PF contributed to extended analgesia and enhanced patient comfort. Overall, this pain management strategy was effective in controlling postoperative pain and resulted in high satisfaction from the patient, family, and surgical team.

## Figures and Tables

**Table 1:** Medication Administration.

	Fentanyl (mcg/kg)	Methadone IV (mcg/kg)	Ketamine (mg/kg)	Hydromorphone (mcg/kg)	Acetaminophen IV or PO (mg/kg)	Ketorolac (mg/kg)
Intraoperatively	2.25	0.22	1.87	0	14.98	0
Postoperative Day 0	0	0.11	0.66	0	14.98	0
POD 1	0	0.22	1.14	5.99	74.9	0
POD 2	0	0.22	0	0	74.9	0
POD 3	0	0.17	0	0	44.94	1.57
POD 4	0	0.06	0	3.75	59.92	2.1
POD 5	0	0.06	0	0	29.96	0.52
POD 6	0	0	0	0	As needed	0

**Table 2:** Postoperative Pain Scores Collected Using the Visual Analog Scale and Mobility.

	Average Pain Score/10	Highest Pain Score/10	Out of Bed in Chair (Y/N)	Ambulation (Y/N)
Postoperative Day 0	0	0	N	N
POD 1	0.5	6	Y	N
POD 2	0.29	2	Y	Y
POD 3	0.4	2	Y	Y
POD 4	1	7	Y	Y
POD 5	0.29	2	Y	Y
POD 6	0	0	Y	Y

Y: yes; N: no.

## Data Availability

The data presented in this study are available on request from the corresponding author due to privacy reasons.

## Supplementary Materials

The following supporting information can be downloaded at https://www.mdpi.com/article/10.3390/children12111450/s1, Video S1: Spread of local anesthetic from blocks performed at different levels.

## Abbreviations

The following abbreviations are used in this manuscript:

AAA	Abdominal aortic aneurysm
PF	Preservative-free
RSB	Rectus sheath block
POD	Postoperative day
IV	Intravenous
NPO	Nil per os
TAP	Transversus abdominus plane
